# The Effectiveness of Interventions in Improving Hand Hygiene Compliance: A Meta-Analysis and Logic Model

**DOI:** 10.1155/2021/8860705

**Published:** 2021-07-17

**Authors:** Mohammad Hossein Kaveh, Mohadeseh Motamed-Jahromi, Soheil Hassanipour

**Affiliations:** ^1^Research Center for Health Sciences, Institute of Health, Department of Health Promotion, School of Health, Shiraz University of Medical Sciences, Shiraz, Iran; ^2^Department of Health Promotion, School of Health, Shiraz University of Medical Sciences, Shiraz, Iran; ^3^Cardiovascular Diseases Research Center, Department of Cardiology, Heshmat Hospital, School of Medicine, Guilan University of Medical Sciences, Rasht, Iran

## Abstract

**Background:**

Despite the availability of various guidelines, rules, and strategies, hand hygiene adherence rates among healthcare workers are reported significantly lower than expected. The aim of this meta-analysis is to determine the most effective interventions to improve hand hygiene and to develop a logic model based on the characteristics of the most effective interventions.

**Methods:**

A literature search was conducted on PubMed, ProQuest, Web of Knowledge, Scopus, Cochrane Library, and ScienceDirect databases up to December 21, 2019, with no time limit. Randomized clinical trials which had designed interventions to improve hand hygiene were reviewed. Data were extracted independently by two authors. All statistical analyses were performed using Comprehensive Meta-Analysis (CMA) software (version 2.0). A random-effects model was used to estimate odds ratios.

**Results:**

Although 14 studies were initially reviewed, only 12 studies entered the meta-analysis, since they had identified percentage rates of hand hygiene compliance. The most effective intervention (odds ratio 18.4, 95% CI (13.6–24.8)) was a multilevel strategy that influenced the determinants of hand hygiene behavior at individual, interpersonal, and organizational levels. Following this, a theory-driven logic model was mapped out to promote hand hygiene, based on situational analysis.

**Conclusion:**

This study suggests that designing integrated interventions based on a multilevel socioecological approach has the greatest potential to improve hand hygiene compliance in healthcare workers. The logical model proposed in this study can thus provide a useful guide for designing and conducting future experimental research.

## 1. Introduction

Hospital infections can pose significant threats to healthcare systems, affecting the rates of illness, mortality, and length of in-hospital stay, while also increasing healthcare costs [1–5]. A global survey conducted by the World Health Organization (WHO) estimates 7%–12% of hospitalized patients suffered from nosocomial infection [6]. It is noteworthy that the relationship between improving hand hygiene and reducing nosocomial infection has been indicated for more than 150 years, and hand hygiene has been widely accepted as the cornerstone of infection prevention and control programs [7–9]. However, the presence of frequent nosocomial infections indicates poor adherence to hand hygiene [10]. According to research, the rate of adherence to hand hygiene among healthcare providers has been estimated at 20–40% [11].

The reasons for poor hand hygiene adherence may include lack of facilities, lack of staffing, busy workplaces, skin irritation, lack of role models, and disregard for instructions [1, 12–14]. Therefore, the strategies for improving hand hygiene adherence should focus on multiple approaches [15, 16]. In this regard, the World Health Organization has provided multiple strategies, including system change, education and training, observation and feedback, hospital reminders, and a safe hospital environment [17].

Previous studies have also employed multifaceted strategies, which have included management and procurement support, education and training, reminders, monitoring, performance feedback [18], empowerment, environmental reconstruction, encouragement [19] and goal setting, reward incentives, and accountability [20]. However, these strategies have had multiple design limitations, which have made it difficult to judge their overall effects. For example, multiple factors influencing adherence to hand hygiene were not initially identified within these studies, and therefore strategies were not systematically tailored based on situational analysis. In addition, most strategies targeted only short-term outcomes.

The success of a multifaceted intervention depends on the success of a complete simultaneous sequence of influencing factors [21]. Accordingly, it is necessary to determine the most effective strategy and its influencing components to increase adherence to hand hygiene. Likewise, it is important to draw a logical model to represent the relationships between a program's components and intended effects. Logical models are implicit maps that convey a plan, program, or project in a concise and visual template [22]. Rational models increase the likelihood of a program succeeding because they determine potential barriers to program implementation, goals, target audiences, required actions for optimal outcomes, and expected outcomes [23]. Therefore, this meta-analysis was conducted to determine the best interventions and then develop a conceptual framework of a logical model, based on the characteristics of effective interventions regarding hand hygiene adherence.

## 2. Materials and Methods

This review was conducted based on the PRISMA checklist (Preferred Reporting Items for Systematic Reviews and Meta-Analysis).

The research questions were as follows:

- What are the most effective interventions for improving hand hygiene compliance?
- How can a logic model be drawn based on the characteristics of effective interventions?

### 2.1. Search Strategy

In order to find interventional studies on improving hand hygiene adherence, PubMed, ProQuest, Web of Knowledge, Scopus, Cochrane Library, and ScienceDirect databases were searched up to December 21, 2019, without a time limit. Additionally, a list of references of similar studies and relevant systematic reviews was examined for further information. The search strategy was developed by the experienced research team (Appendix).

The keywords used in the initial search were (Compliance OR adherence) AND (Nurses OR healthcare worker OR healthcare practitioner OR infection control worker OR infection control practitioner OR infection control staff OR infection control personnel OR healthcare provider) AND (hand hygiene OR hand wash^∗^).

The collected data were entered in EndNote X7 software and duplicate articles were removed automatically. It is worth noting that two researchers examined the articles separately.

### 2.2. Eligibility Criteria

All studies with the following criteria were included: (a) randomized controlled trials (RCT), in which at least one intervention had been performed to improve healthcare provider compliance to hand hygiene; (b) studies that determined the level of hand hygiene compliance; and (c) studies conducted on health practitioners working at hospitals. The exclusion criteria were as follows: (a) abstracts without full text, (b) abstracts and congress and conference reports in nonarticle structure, (c) studies that were not original articles, and (d) non-English language studies.

### 2.3. Extracting Data

An initial search for studies was conducted by two authors of this study (M. MJ and S. H). Screening, extracting the results, and assessing the quality were carried out by these two individuals separately. In case of inconsistency between the two judgments, the supervisor determined the final comment on that article (MH. K).

### 2.4. Risk of Bias among Studies

A random-effects model was used to reduce the risk of bias in studies. The Egger test and funnel plot were used to examine publication bias.

### 2.5. Statistical Analysis

Heterogeneity among studies was measured using Cochran's test (with significance level <0.1) and their combination was examined using *I*^2^ statistics. In the case of heterogeneity, a random-effects model with the inverse-variance method was applied. All analyses were performed using Comprehensive Meta-Analysis (CMA) software version 2.

### 2.6. Effect Size

The effect size used in this study was the calculated odds ratio (OR) index. Odds ratio indicator allows the different results to be combined and presented in a comprehensible way. CMA software has the ability to combine different indices, as well as combining the effect of the sample size and the difference of the index being compared. Thus, the reasons for using the odds ratio were the different indicators, measurement scales, and data analysis methods based on the subgroups in the studies.

The calculated OR eliminates all these restrictions and provides a more meaningful indicator. The calculated OR can be interpreted in the following way: if the OR is greater than one, the intervention is beneficial. If the OR is equal to 1, the odds of one event are the same for both groups, while if the OR is less than one, the intervention has a reverse effect.

### 2.7. Development of a Logic Model

Finally, a logic model was created and developed based on the characteristics of effective interventions to provide a conceptual framework for the improvement of hand hygiene adherence. A logic model clarifies the logic of a program in terms of achieving its goals and objectives [24]. The components of a logic model consist of inputs, processes, and outcomes [25]. Inputs are the resources that will later be transformed into outcomes and should be specified as part of planned activities [26]. The process demonstrates how the theories, methods, and activities of the planned program act in service of the goal with regard to the participants [27]. Outcomes illustrate what the program achieves in terms of knowledge, attitudes, and skills among participants [28].

## 3. Results

### 3.1. Quality Assessment

**Figure 1:**
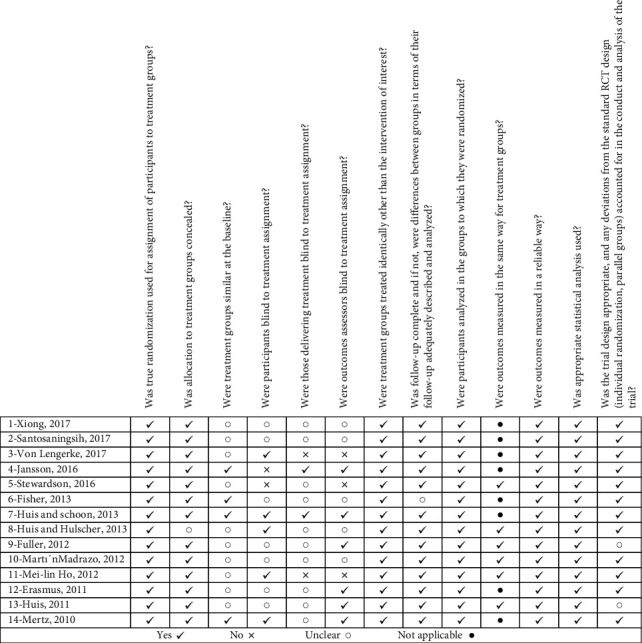
Assessment risk of bias in included studies of randomized clinical trial studies to promote hand hygiene of healthcare workers.

The checklist for a randomized controlled trial prepared by the Joanna Briggs Institute (JBI) [29] was used for quality assessment. The studies were assessed using the checklist items.  Figure 1 shows the quality of the articles.

#### 3.1.1. Search Description

**Figure 2:**
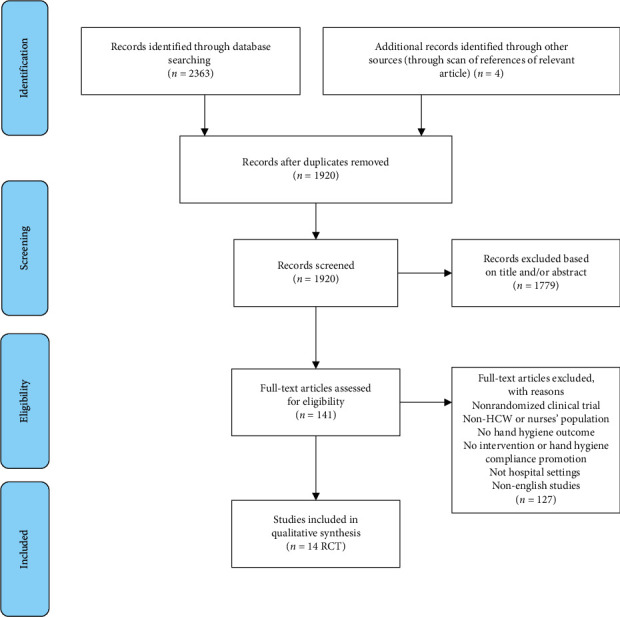
Search process and study identification systematic review of randomized clinical trial studies to promote hand hygiene in healthcare workers.

After searching all international databases, 2363 articles were initially found. After removing duplicate articles, 1920 articles were examined in terms of topic and abstract, out of which 141 articles entered the next stage. At this stage, the full texts of the articles were examined, and 14 RCT articles entered the final analysis. It should be noted that the references of the articles included were also reviewed to add relevant studies. In the screening stage, some studies were excluded for several reasons, due to irrelevant topics and irrelevant study populations. The flowchart of the included studies is presented in Figure 2.

#### 3.1.2. Characteristics of the Studies Included

14 randomized clinical trials were found by the researchers, of which 4 studies were conducted in the Netherlands, while the rest were conducted in China, Germany, Indonesia, Sweden, Finland, Hong Kong, Spain, England, Canada, and the United States [30–43]. Of these, 12 studies were included in the meta-analysis, since they had identified the percentage of hand hygiene compliance. The characteristics of the randomized clinical trials included in the meta-analysis are presented in Table 1.

### 3.2. Meta-Analysis and Data Synthesis

#### 3.2.1. Heterogeneity

The result of the chi-squared test and the *I*^2^ index indicated that there was considerable between-study heterogeneity. Due to the high heterogeneity in results, the random effect model was used to estimate the overall odds ratio.

### 3.3. Synthesis of Results

**Figure 3:**
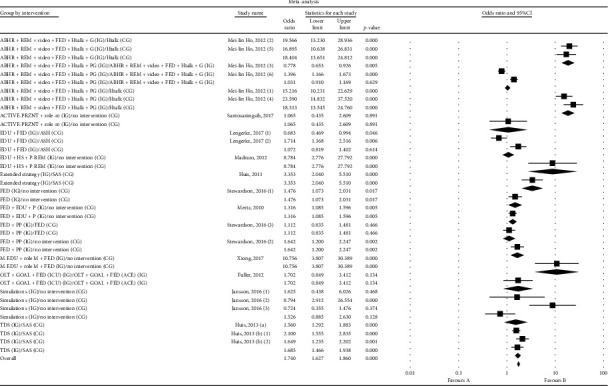
Forest plot accumulation curve: the effect of interventions compared to other groups (95% confidence interval).

In this study, the overall odds ratio was 1.74 with a 95% confidence interval (1.62–1.86), *P* < 0.001. The results of the meta-analyses are displayed in Figure 3.

The results showed that the multimodel strategy (alcohol-based hand rub + video + reminders + feedback + health talk + powerless gloves) conducted by Ho et al. [33], with a higher odds ratio and smaller variation span, was the most effective intervention in terms of hand hygiene adherence (OR = 18.4, 95% CI (13.6–24.8), *P* < 0.001, *I*^2^ = 95.8%, *P* < 0.001).

In a study conducted by Martín-Madrazo et al. [38], the intervention group which involved training sessions, the application of hydroalcoholic solutions, and setting reminder posters showed greater effectiveness than the control group (OR = 8.78, 95% CI (2.7–27.7), *P* < 0.001, *I*^2^ = 99.1%, *P* < 0.001).

In Huis et al.'s study in 2011 [35], an extended strategy targeting social impact and sustained leadership in the intervention group showed more effectiveness, compared with a control group (OR = 3.35, 95% CI (2.04–5.51), *P* < 0.001, *I*^2^ = 95.8%, *P* < 0.001).

In another study performed by Stewardson et al. [41], the intervention group that used sustained performance feedback and patient participation showed more effectiveness compared to a control group (OR = 1.47, 95% CI (1.07–2.03), *P*=0.01, *I*^2^ = 95.2%, *P* < 0.001).

The research conducted by Mertz et al. [39] revealed that the intervention group with performance feedback and small group educational seminars showed more effectiveness than the control group (OR = 1.31, 95% CI (1.08–1.59), *P*=0.005, *I*^2^ = 88.0%, *P* < 0.001).

Xiong et al. [43] indicated that multimedia training sessions, video presentation, role-playing, and feedback in the intervention group were more effective in comparison with self-learning in the control group (OR = 10.75, 95% CI (3.8–30.3), *P* < 0.001, *I*^2^ = 99.3%, *P* < 0.001).

Huis et al. conducted two studies in 2013 [34, 36]. A team and leaders-directed (TDS) strategy were used in the intervention group of both studies and eventually, the intervention group showed a significantly improved effectiveness, compared with the control group (OR = 1.6, 95% CI (1.46–1.93), *P* < 0.001, *I*^2^ = 85.9%, *P* < 0.001).

Finally, the results of the intervention groups in four studies included an intervention using active presentation and role modeling [40], a tailored intervention based on health action process approach (HAPA) [42], an intervention that included simulated sessions [37], and a feedback intervention using goal setting and control and operant-learning theories [32] did not show a statistically significant difference compared with the control groups.

#### 3.3.1. Assessing Publication Bias

**Figure 4:**
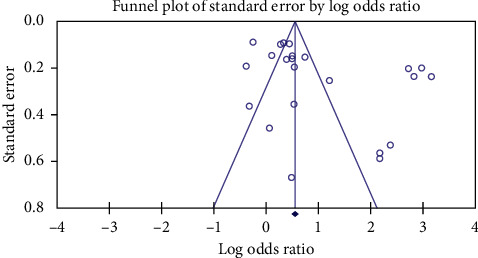
Funnel plot of included studies.

Based on the results of Egger's test, publication bias was observed among the studies (*P* < 0.001).  Figure 4 shows the funnel plot of the studies.

## 4. Discussion

The main aim of the present meta-analysis was to determine the most effective intervention, as well as its most influential components, in improving hand hygiene. In analyses stratified by type of intervention among 9 RCTs with effective strategies, a multimodel strategy in the study of Ho et al. was recognized as the most effective intervention, affecting three levels: individual, interpersonal, and organizational. In this intervention, provision of facilities, health talk, train-the-trainer approach, feedback, reminders, and performance reports were seen to work effectively [33]. Schölmerich and Kawachi, in line with this finding, stated that multilevel interventions inspired by socioecological models enhance the impact of programs by simultaneously altering the individual, social, and organizational levels [44].

According to the findings, another effective strategy, which had an impact on both individual and organizational levels, involved providing facilities, training, and reminder posters [38]. Naikoba and Hayward confirmed that strategies including supplemental interventions such as education, reminders, and giving feedback are more useful than strategies that only provided more facilities [45].

Several successful studies using a team and leaders-directed strategy affected two levels: interpersonal (social influence) and organizational (sustained leadership and managers) [34, 36]. The evidence suggests that team-based strategies can be effective in improving hand hygiene adherence [36, 46]. Huis et al. stated that team-based strategies emphasize social influence, team effectiveness, and leadership theory as well as providing a way to develop hand hygiene behavior at the interpersonal and organizational levels [36].

Based on the results, performance feedback (organizational level) in conjunction with feedback involving patient participation (interpersonal level) was another successful strategy [39]. Jansson et al. similarly presented a successful strategy of sustained feedback and small group training seminars at organizational and interpersonal levels [37]. Several studies in line with this finding explained that patient requests for hand hygiene compliance, along with continuous performance feedback (immediate verbal feedback, giving hand hygiene observation cards, and personalized advice by supervisors and managers) can have a significant impact on hand hygiene adherence [41, 47, 48].

Another strategy involving mixed-media education and feedback had a significant impact on promoting hand hygiene adherence at both interpersonal and organizational levels [41]. It seems that using mixed-media education can improve the individuals' capacity to use learned theoretical concepts to solve real-world problems [49]. Consistent with this finding, Chun et al. believed that various methods of education about hand hygiene, linked with subsequent feedback, would improve the frequency and quality of hand hygiene activities and will also increase awareness [50].

### 4.1. A Proposed Logic Model for Designing Interventions

According to the results, there is a missing point in designing the successful strategies; they did not pay attention to situational analysis and therefore the interventions were not systematically designed. Situation analysis is important because it refers to information gathering and analysis of the context's current state in terms of physical conditions, material, human resources, and so forth, which can have a significant impact on the selection of appropriate and accurate strategies [51]. Since logic models are designed based on situational analysis, the authors of this paper decided to propose a logic model for improving hand hygiene compliance. In fact, a logic model is a simplified visual representation of the typology of interventions; processes; and short-term, medium-term, and long-term outcomes that can be a suitable platform for a realistic systematic review [52–54].

The proposed logic model was designed based on a socioecological approach (individual, interpersonal, and organizational levels). In the first step, a situational analysis of the current state of the context in terms of hand hygiene behavior in employees, group norms, physical environment characteristics, nosocomial infection rate, and organizational dynamics (resources, organizational policies, and leadership style) is performed (Table 2).

In the input part of Table 2, it is specified that, to improve hand hygiene, interventions should be tailored for the target groups in the clinical environment, including healthcare workers, patients, supervisors, and decision-makers. The change objectives should be also determined in relation to the target groups.

In the process section, at the individual level, it is important to use different activities to improve knowledge, perceptions, and attitudes towards hand hygiene compliance. The best theories for planning activities at this level involve using empowerment-oriented behavior change theories, such as Theory of Planned Behavior (TPB) [55] and Freire's model of adult education [56].

At the interpersonal level, activities may be planned according to social norms theory [57, 58], while organizational change theory [59] may be used at the organizational level. In the activities section, several activities based on theory and methods are suggested for planners. Short-term outcomes show the effect of interventions on attitudes, perceptions, knowledge, and beliefs; changes in behavior, decision-making, and action are considered as medium-term outcomes; long-term outcomes involve major changes in status [60]. These outcomes are suggested based on the hand hygiene topic in the logic model.

## 5. Limitations

One of the most important limitations of this study was the existence of different reports on the effectiveness of each intervention, which made it difficult to combine the studies. However, combining the results was made possible by using the capabilities of CMA software. A second limitation was reviewing only studies that measured hand hygiene adherence as a percentage via behavioral observation so that the studies were excluded involving self-reporting and assessing the quality of the process. The last limitation was that almost all studies were conducted at well-equipped hospitals in developed countries, so generalization of the results to other hospitals or less developed countries should be made with caution.

## 6. Conclusions

The present meta-analysis identifies that the use of a socioecological approach in planning comprehensive and coordinated interventions (which target behavioral determinants at multiple levels of influence) is significantly more effective than other multiple-level strategies. Therefore, the authors of this study present a new theory-based logic model that can be an appropriate guide for planning, implementing, and evaluating an intervention at these three levels. It is suggested that decision-makers, planners, and managers in hospitals use this proposed logic model for setting up interventions to improve hand hygiene so that they can design a comprehensive and effective intervention. Future studies can be performed to determine the effectiveness of the proposed logic model in the field and different healthcare scenarios.

## Figures and Tables

**Table 1:** Characteristics of the randomized clinical trials included in the meta-analysis.

Author	Year/country	Design	Sample	Groups	Intervention package	Baseline-follow-up intervals (month)	Compliance%				
							Baseline		Follow-up 1	Follow-up 2	Follow-up 3
*Xiong et al.*	2017/China	CRCT	40 nurses	Control	No intervention	0, 1.5	CG	61.64	62.63		
			40	Intervention	M-EDU + role M + FED		IG	62.67	72.55		

*Von Lengerke et al.*	2017/Germany	CRCT	405 HCW	Control	ASH	0, 12, 24	CG	54.00	68.00	64.00	
			682	Intervention	EDU + FED		IG	54.00	64.00	70.00	

*Santosaningsih et al.*	2017/Indonesia	CRCT	62 HCW	Control	No intervention	0, 2	CG	10.10	20.50		
			284	Intervention	Active-presentation + Role-m		IG	16.10	27.10		

*Stewardson et al.*	2016/Switzerland	CRCT	21 wards	Control	No intervention	0, 24	CG	66.00	73.00		
			24	Intervention 1	FED		IG	65.00	75.00		
			22	Intervention 2	FED + PP		IG	66.00	77.00		

*Jansson et al.*	2016/Finland	RCT	15 nurses	Control	No intervention	0, 3, 6, 24	CG	43.30	39.10	45.80	56.60
			15	Intervention	Simulation session		IG	40.80	38.30	59.20	50.80

*Huis and Schoon et al.*	2013/Netherlands	CRCT	1083 nurses	Control	SAS	0, 6	CG	21.80	45.90		
			1083	Intervention	TDS		IG	19.10	52.10		

*Huis and Hulscher et al.*	2013/Netherlands	CRCT	518 nurses	Control	SAS	0, 6, 12	CG	23.00	42.00	46.00	
			415	Intervention	TDS		IG	20.00	53.00	53.00	

*Mei-lin Ho et al.*	2012/Hong Kong	CRCT	942 staff and resident	Control	Htalk	0, 1, 4	CG	19.50	19.80	21.60	
			1015	Intervention 1	ABHR + REM + video + FED + Htalk + PG		IG	27.00	59.20	60.60	
			1260	Intervention 2	ABHR + REM + video + FED + Htak + G		IG	22.20	59.90	48.60	

*MartinMadrazo et al.*	2012/Spain	CRCT	99 HCW	Control	No intervention	0, 6	CG	8.26	11.86		
			99	Intervention	EDU + HS + P-REM		IG	7.98	32.74		

*Fuller et al.*	2012/England	CRCT	16 wards	ICU	OLT + GOAL + FED	0, 6	IG	13.00	18.00		
			44	ACE	OLT + GOAL + FED		IG	10.00	13.00		

*Huis et al.*	2011/Netherlands	CRCT	450 nurses	Intervention 1	SAS	0, 6	IG	10.00	15.00		
			450	Intervention 2	Extended strategy		IG	10.00	25.00		

*Mertz et al.*	2010/Canada	CRCT	15 wards	Control	No intervention	0, 6	CG	15.90	42.60		
			15	Intervention	FED + EDU + P		IG	15.80	48.20		

CRCT = cluster randomized clinical trial; HCW = healthcare worker; m = month; Role M = role model training.; EDU = education; M-EDU = media education; FED = feedback; ASH = Aktion Saubere Hände (Clean Hands Campaign); PP **=** patient participation; SAS = state-of-the-art strategy; TDS = team leaders-directed strategy; Htalk = health talk; ABHR = alcohol-based hand rub; PG = powered gloves; G = gloves; HS = hydroalcoholic solutions; REM = reminders; OLT = operant-learning theories; P **=** posters; GOAL = goal setting; CG = control group; IG = intervention group.

**Table 2:** A logic model based on a socioecological approach delineating inputs, processes, and outcomes to improve hand hygiene compliance. 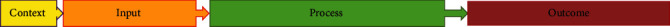

Situational analysis	Intervention target	Target audience	Change objectives	Theory	Methods	Activities	Short-term outcomes	Medium-term outcomes	Long-term outcomes
	Influence individuals	Healthcare workers (HCWs), patients	Hand hygiene compliance	(i) Theory of Planned Behavior (TPB) (ii) Freire's model of adult education	(i) Discussion (ii) Problem-based learning (iii) Guided exploration	(i) Identifying the advantages and disadvantages of performing hand hygiene (ii) Determining people whose approval is important for the person to do hand hygiene (iii) Identifying barriers to performing hand hygiene (iv) Identifying facilitators to performing hand hygiene (v) Brainstorming root causes of poor hand hygiene adherence (vi) Personalizing the issue and using role-plays to generate emotions	(i) Improved knowledge, perception, and attitudes about hand hygiene compliance	(i) Compliance with the WHO “5 moments of hand hygiene” responsibly (ii) Sustained improvement in hand hygiene	(i) Reduced nosocomial infections
	Influence interpersonal level	Coworkers and supervisors	Supportive behavior Emotional, informational, appraisal, and instrumental support	(i) Social norms theory	(i) The train-the-trainer method	(i) Participatory discussions between HCWs about hand hygiene compliance (ii) Creation of new knowledge in a selected group of participants (iii) Sharing knowledge with others	(i) Improved social norms about hand hygiene in workplace	(i) Social approval for hand hygiene compliance (ii) HCWs imitate each other in performing hand hygiene (iii) Sustained improvement in hand hygiene	
	Influence organizational level	Decision-makers	Supportive environment: policies and regulations	Organizational change theory	(i) Planning (ii) Monitoring (iii) Reviewing (iv) Rewarding	(i) Designing new and innovative policy for improving hand hygiene (ii) Providing the essential materials and equipment for hand hygiene compliance (iii) Continuous monitoring of hand hygiene compliance among staff and patients (iv) Appropriate reaction to hand hygiene behavior at the right time and place (v) Performance review with employee self-assessment (vi) Encouragement of wards that have the highest hand hygiene and the lowest nosocomial infections	(i) Responsive policy for improving hand hygiene	(i) Sustained leadership (ii) Continuous evaluation (iii) Sufficient facilities (iv) Sustained improvement in hand hygiene	

## Data Availability

This manuscript is a systematic review and meta-analysis. Different tables used to support the findings of this study are included in the article. The search strategy process used to support the findings of this study is included within the supplementary information file. Requests for access to the endnote file should be sent to Mohadeseh Motamed-Jahromi, e-mail: mohadesehmotamed@yahoo.com.

## Supplementary Materials

Supplementary Materials Appendix Aa: search strategies in PubMed, ProQuest, Web of Knowledge, Scopus, Cochrane Library, and ScienceDirect database.
