# Supplementary material for: The Effectiveness of Interventions in Improving Hand Hygiene Compliance: A Meta-Analysis and Logic Model
Source: Can J Infect Dis Med Microbiol. 2021 Jul 17;2021:8860705. doi: 10.1155/2021/8860705 (PMC8313351; doi:10.1155/2021/8860705)
Supplement: Supplementary Materials — Appendix Aa: search strategies in PubMed, ProQuest, Web of Knowledge, Scopus, Cochrane Library, and ScienceDirect database. [file 8860705.f1.docx]

# Search strategy:

**All times Until December 21th, 2019**

| **(PubMed)** | |  |
| --- | --- | --- |
| 1 | (compliance[Title/Abstract]) OR adherence[Title/Abstract] Filters: Full text | [186825](https://www.ncbi.nlm.nih.gov/pubmed/?cmd=HistorySearch&querykey=5) |
| 2 | \| (((((((Nurses[MeSH Terms]) OR healthcare worke[Title/Abstract]) OR healthcare practitioner[Title/Abstract]) OR infection control worker[Title/Abstract]) OR infection control practitioner[Title/Abstract]) OR infection control staff[Title/Abstract]) OR infection control personel[Title/Abstract]) OR healthcare provider[Title/Abstract] Filters: Full text \| \| --- \| | [50368](https://www.ncbi.nlm.nih.gov/pubmed/?cmd=HistorySearch&querykey=7) |
| 3 | (hand hygiene[MeSH Terms]) OR hand wash*[Title/Abstract] Filters: Full text | [6173](https://www.ncbi.nlm.nih.gov/pubmed/?cmd=HistorySearch&querykey=8) |
| 4 | \|  \| #1 AND #2 AND #3  (((((compliance[Title/Abstract]) OR adherence[Title/Abstract]) AND full text[sb])) AND (((((((((Nurses[MeSH Terms]) OR healthcare worke[Title/Abstract]) OR healthcare practitioner[Title/Abstract]) OR infection control worker[Title/Abstract]) OR infection control practitioner[Title/Abstract]) OR infection control staff[Title/Abstract]) OR infection control personel[Title/Abstract]) OR healthcare provider[Title/Abstract]) AND full text[sb])) AND (((hand hygiene[MeSH Terms]) OR hand wash*[Title/Abstract]) AND full text[sb]) Filters: Full text \| \| --- \| --- \| | 70 |

| **Prequest** | |  |
| --- | --- | --- |
| Additional limits: Fulltext/English/Article | ab(adherence and compliance) AND ab(nurse OR healthcare worker OR healthcare practitioner OR infection control worker OR infection control practitioner OR infection control Staff infection control personnel OR healthcare provider) AND ab(hand hygiene OR hand wash*) | 22 |

| **Web of science** | |  |
| --- | --- | --- |
|  | \|  \| ((TI=((compliance OR adherence) AND (nurse OR healthcare worker OR healthcare practitioner OR infection control worker OR infection control practitioner OR infection control Staff infection control personnel OR healthcare provider) AND (hand hygiene OR hand wash*)))) AND LANGUAGE: (English) AND DOCUMENT TYPES: (Article) \| \| --- \| --- \| | 43 |

| **Scopus** | |  |
| --- | --- | --- |
|  | ( TITLE-ABS-KEY ( compliance ) OR TITLE-ABS-KEY ( adherence ) AND TITLE-ABS-KEY ( nurs* ) OR TITLE-ABS-KEY ( healthcare AND worker ) OR TITLE-ABS-KEY ( healthcare AND practitioner ) OR TITLE-ABS-KEY ( infection AND control AND worker ) OR TITLE-ABS-KEY ( infection AND control AND practitioner ) OR TITLE-ABS-KEY ( infection AND control AND staff ) OR TITLE-ABS-KEY ( infection AND control AND personnel ) OR TITLE-ABS-KEY ( healthcare AND provider ) AND TITLE-ABS-KEY ( hand AND hygiene ) OR TITLE-ABS-KEY ( hand AND wash* ) ) AND DOCTYPE ( ar ) AND ( LIMIT-TO ( LANGUAGE , "English" ) ) AND ( LIMIT-TO ( SRCTYPE , "j" ) ) | 1466 |

| **Cochrane Trials Register** | |  |
| --- | --- | --- |
|  | hand wash or hand washing or hand washed or hand washes or hand hygiene in Title Abstract Keyword AND nurses or health worker or health practitioner or infection control worker or infection control practitioner or infection control staff or infection control personnel or healthcare provider in Title Abstract Keyword AND compliance or adherence in Title Abstract Keyword - in Trials (Word variations have been searched) | 189 |

| **Science Direct** | |  |
| --- | --- | --- |
| filter: Research articles | Title, abstract, keywords: (hand hygiene OR hand wash*) AND (compliance OR adherence) | 573 |

# Extracted papers

**Studies identified through international database search and reference check = 2363**

**Local database search and other sources=4**

**Duplicate finding = 447**

**Studies remained after removal of the duplicates =1920**
